# Enantioselective Magneto-Chiral Photochemistry Rediscovered

**DOI:** 10.1021/acscentsci.5c00772

**Published:** 2025-06-16

**Authors:** Maria Sara Raju, Maxime Aragon-Alberti, Kevin Cardenas, Ivan Breslavetz, Geert L. J. A. Rikken, Cyrille Train, Matteo Atzori

**Affiliations:** Laboratoire National des Champs Magnétiques Intenses, CNRS, Université Grenoble Alpes, INSA Toulouse, Université Toulouse Paul Sabatier, EMFL, F-38042 Grenoble, France

## Abstract

Enantioselective magneto-chiral photochemistry (MChPh), which represents the ability of an unpolarized light beam ***k*** applied along a magnetic field ***B*** to produce an enantiomeric excess (*ee*), was experimentally demonstrated for the first time 25 years ago. Despite the relevance that this effect can have for the origin of molecular homochirality, no other experiment has been reported in the literature since then. With the aim of reexploring this enantioselective photochemical reactivity and quantitatively determining the *ee* achievable through MChPh as a function of the applied magnetic field and the laser irradiation wavelength, we report here on new magneto-chiral dichroism (MChD) studies and MChPh experiments on potassium tris­(oxalato)­chromate­(III). By irradiating a racemic mixture of enantiomers in solution (*T* = 5 °C) at λ = 695.5 nm (500 mW), the wavelength where MChD is maximum, under a magnetic field ***B*** = 30 T for 30 min, an *ee* of 0.50% has been obtained. We demonstrate that under the same experimental conditions, circularly polarized photochemistry (CPPh), the most accredited mechanism at the origin of molecular homochirality, yields a lower *ee*.

## Graphical abstract

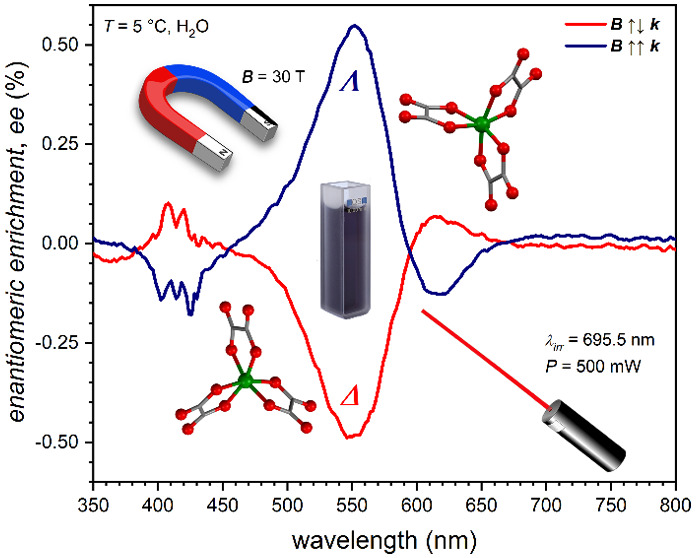

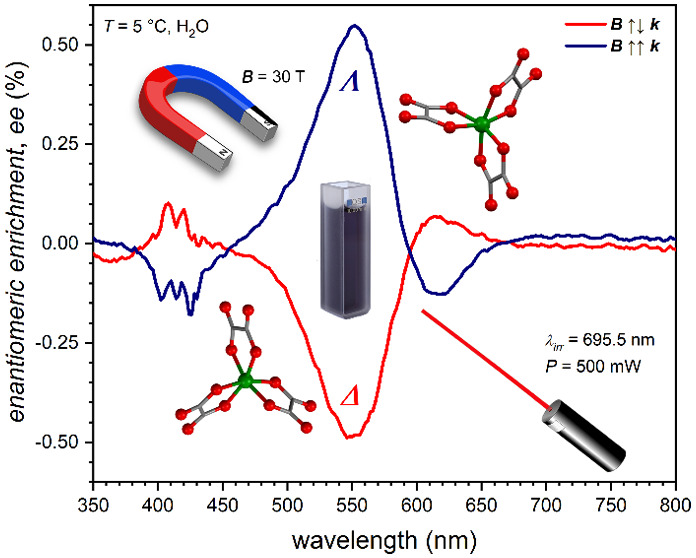

## Introduction

The single-handedness of naturally occurring sugars and amino acids, the molecular building blocks of biological polymers, is a signature of life. This unique property is considered a prerequisite for the origin or early evolution of life because it is crucial for the efficiency of molecular recognition and self-organization, hence enzymatic functions and structural arrangement, of biological systems.^1–4^ Nonetheless, in the absence of a chiral driving force, an abiotic process commonly yields an equal mixture of left- and right-handed molecules. Therefore, to reach homochirality, specific physical processes, chemical reactions, or a combination of them is necessary to generate an initial enantiomeric excess (*ee*) of molecular building-blocks of a given handedness.^1–4^

The process toward molecular homochirality is thought to proceed in two steps.^1,2^ The first step consists of obtaining an *ee*, as tiny as it can be, starting from achiral systems or racemic mixtures. The second step involves the amplification of this *ee* to allow the development of a fully homochiral biological life.^3,5^

Photochemistry with circularly polarized light (CPL) represents one of the most accredited and studied mechanism for the generation of molecular *ee*.^4,6,7^ CPL has an intrinsic chiral nature (left- and right-handed) that makes it able to enantioselectively interact with chiral molecules, as exemplified by the phenomenon of natural circular dichroism (NCD). Because the interaction between CPL of one handedness with the two enantiomers of a chiral chromophore is not equivalent, the irradiation of a racemic mixture of chromophores with CPL can generate an *ee*. For example, the circularly polarized light photochemistry (CPPh) of racemic leucine at λ = 213 nm yields an *ee* of ca. 2–3%, the predominant enantiomer depending on the light handedness.^8,9^

It should be noted, however, that natural sources of light are essentially unpolarized. On Earth, small amounts of CPL are produced from our Sun, while in space, non-negligible amounts of CPL were detected in regions composed of clouds of gas and plasma where stars formation takes place, and organic molecules generated from CO, CO_2_, and CH_3_OH precursors are abundant.^2,6^ The largest degrees of CPL are observed in the near-infrared spectral window (900–2200 nm),^2^ which is not the most effective energy range to induce photochemical reactions involving prebiotic molecules such as amino acids, that are mainly driven by ultraviolet (UV) light.^1,8,9^

However, there exists a polarization independent mechanism that through the combination of unpolarized light and magnetic fields has the same enantioselective features as NCD. This is called magneto-chiral dichroism (MChD) and manifests as an enantioselective difference in the absorption of unpolarized light by chiral systems in a magnetic field.^10–12^ More specifically, a magnetic field ***B*** applied along the direction of an unpolarized light beam ***k***, causes a modification of the absorption coefficient of a chiral molecule, which is equal in magnitude but opposite in sign for the two enantiomers. Therefore, the combined effect of these two physical entities induces a true chiral influence on chiral systems similar to CPL, as elegantly demonstrated by L. Barron in 1986.^13,14^ Contrary to CPL, unpolarized light and magnetic fields are abundant and ubiquitous in our universe. However, to provide a net effect, their relative orientation needs to fulfill the above-mentioned conditions. In outer space, huge magnetic fields and intense unpolarized light are generated by supernovae, which provide favorable conditions for the generation of an *ee* through unpolarized light irradiation in a magnetic field, a phenomenon called enantioselective magneto-chiral photochemistry (MChPh), on organic matter in nearby interstellar dust clouds.

The implication of MChD in MChPh was demonstrated by one of us in 2000 through the photoresolution of the tris­(oxalato)­chromate­(III) complex using an unpolarized laser beam.^15–17^ A clear *ee* was observed at relatively low magnetic fields, and the effect could be semiquantitatively understood and modeled.^16^ Despite its remarkable implications, this MChPh experiment remains, to the best of our knowledge, the only one reported to date.

To deeply explore MChPh and give new inputs to this research field by using modern experimental setups and higher magnetic fields, we have investigated the photoresolution of a racemic mixture of (*Λ*)- and (*Δ*)*-*tris­(oxalato)­chromate­(III) under different experimental conditions and with applied magnetic fields up to 30 T. Moreover, similar experiments of CPPh at the same wavelengths and laser intensity in the absence of a magnetic field were done to allow for a direct comparison of the efficiency of the two mechanisms in generating an *ee*.

### Results and Discussion

The tris­(oxalato)­chromate­(III) complex is an archetypal chiral coordination complex with helical chirality at the metal center. It can be obtained by reducing and coordinating potassium dichromate­(VI) with a mixture of oxalic acid and potassium oxalate in water solution.^18^ The three bidentate oxalate ligands coordinate the Cr­(III) ion in two different left- or right-handed dispositions, stereochemically identified as (*Λ*) and (*Δ*) enantiomers, respectively (Figure S1).^19^ In solution, these two forms coexist as a racemic mixture. In water, the equilibrium between the two forms is ensured by a dissociation/reassociation mechanism, which is accelerated by light absorption. In the solid state, it crystallizes as a trihydrate potassium salt, K_3_[Cr­(C_2_O_4_)_3_]·3H_2_O.^18^ Racemic potassium tris­(oxalato)­chromate­(III) ((*rac*)-**1** hereafter) can be chemically separated in the two enantiopure forms ((*Λ*)-**1** and (*Δ*)-**1**) by second-order asymmetric synthesis using enantiopure tris­(1,10-phenantroline)­nickel­(II) chiral cations.^20,21^

The optical properties of **1** in solution are well-known. Therefore, we recall here only the most salient features. The absorption spectrum (350–800 nm) is characterized by three main absorption bands associated with two spin-allowed (^4^A_2_ → ^4^T_1_, λ_max_ = 422 nm; ^4^A_2_ → ^4^T_2_, λ_max_ = 572 nm) and one spin-forbidden (^4^A_2_ → ^2^T_1_,^2^E, λ_max_ 698.5 nm) *d-d* electronic transitions (Figures 1 and S2).^19^ These latter transitions, where the excited states differ from the ground state only by the spin multiplicity, are also called spin-flip transitions.^22^

**Figure 1:**
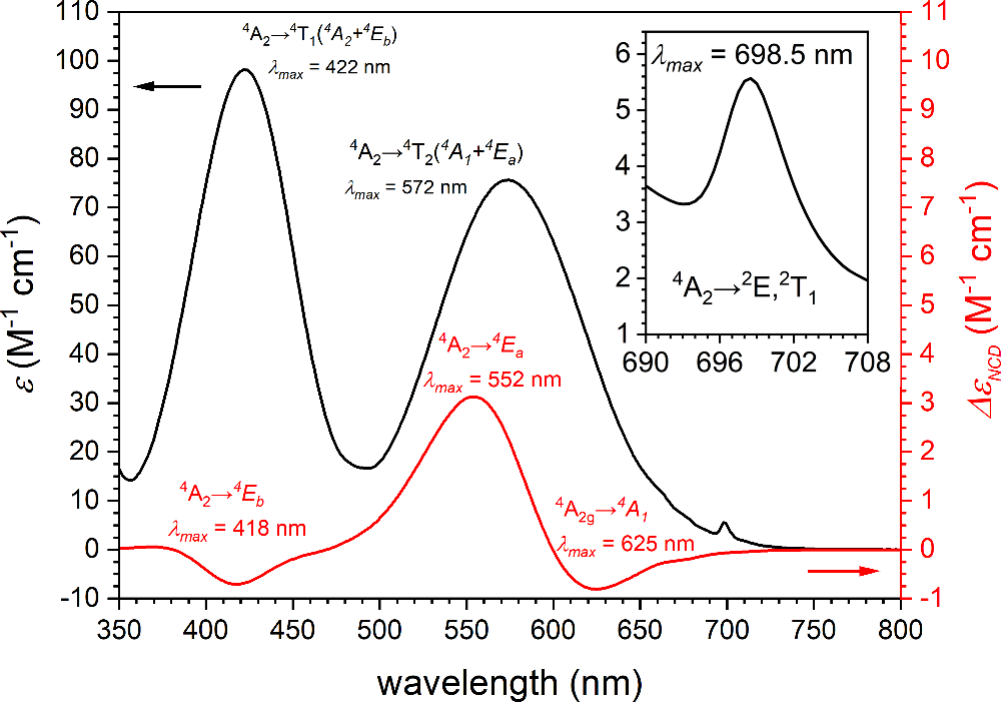
Absorption (black) and NCD (red) spectra of (*Λ*)-**1** in DMSO solution (see legend) reported as extinction coefficients and differential extinction coefficients versus irradiation wavelength. The electronic transitions associated with the absorption and NCD spectra are indicated. Inset shows a zoom of the 690–708 nm region.

The chiroptical properties of the pure enantiomers (*Λ*)-**1** and (*Δ*)-**1** obtained by chemical resolution (see above) were studied by natural circular dichroism (NCD) spectroscopy in solution at room temperature. Figure 1 reports the NCD spectrum for (*Λ*)-**1**. *Δε*_NCD_ assumes values of −0.70(1) M^–1^ cm^–1^, + 3.13(1) M^–1^ cm^–1^, and −0.80(1) M^–1^ cm^–1^ for the ^4^A_2_ → ^4^E_*b*_ (λ_max_ = 418 nm), ^4^A_2_ → ^4^E_*a*_ (λ_max_ = 552 nm), and ^4^A_2_ → ^4^A_1_ (λ_max_ = 625 nm) transitions respectively, while the *Δε*_NCD_ for the ^4^A_2_ → ^2^E,^2^T_1_ spin-forbidden transition is small (ca. −0.01 M^–1^ cm^–1^) and cannot be easily deconvoluted from the tail of the contribution associated with the ^4^A_2_ → ^4^A_1_ transition (Figure 1). This introduces an uncertainty in the determination of the *g*_NCD_ factor for this transition (see below). Recent studies have demonstrated that a better estimation of the chiroptical activity of this transition can be obtained by circularly polarized light emission studies.^23^

A better view of the relative magnitude of the NCD response with respect to the originating absorption bands is provided by the *g*_NCD_ dissymmetry factor (Figure S3 and Equation S1). The obtained values are in very good agreement with the literature findings.^18^ It should be noted that the wavelength dependence of *g*_NCD_ shows a minimum at λ = 698.5 nm. This is due to a non-negligible absorption coefficient for the ^4^A_2_ → ^2^E, ^2^T_1_ transition and a small NCD response that is difficult to estimate because of the superposition with the tail of the more intense high energy band (see above). However, the NCD response of the spin-forbidden band is weak with respect to those of the other transitions.

Magnetic circular dichroism (MCD) spectroscopy was used to better evaluate the effect of the magnetic field on the electronic transitions of **1**. The room temperature MCD spectrum of (*rac*)-**1** in solution obtained under a static magnetic field ***B*** = 1.6 T is reported in Figure S4 and compared with the NCD spectrum. It shows two sharp contributions of *Δε*_MCD_ = +5.2 × 10^–3^ M^–1^ cm^–1^ T^–1^ and +4.8 × 10^–3^ M^–1^ T^–1^ at λ_max_ = 696.0 and 657.0 nm, respectively, plus an additional sharp contribution of −7.5 × 10^–3^ M^–1^ cm^–1^ T^–1^ at λ_max_ = 483.5 nm. These findings are in agreement with previous studies.^24^ The *g*_MCD_ dissymmetry factors (Equation S2) are plotted in Figure S5. The strongest *g*_MCD_ value, which accounts for ca. 1 × 10^–3^ T^–1^, is observed for the ^4^A_2_ → ^2^E, ^2^T_1_ spin-forbidden transitions (λ = 690–703 nm), which are characterized by a small *g*_NCD_.

Visible light absorption and magneto-chiral dichroism (MChD) spectroscopy on (*Λ*)-**1** dispersed in a KBr pellet were done to determine the *g*_MChD_ dissymmetry factors for the above-mentioned electronic transitions. The low temperature (*T* = 4.0 K) solid-state absorption (450–800 nm range) shows the two expected contributions associated with the ^4^A_2_ → ^4^T_2_ and ^4^A_2_ → ^2^T_1_, ^2^E transitions without any remarkable variation in the absorption maxima with respect to the solution spectra at room temperature (Figure 2a). The ^4^A_2_ → ^2^T_1_, ^2^E transition is well-defined, and the contributions between 650 and 675 nm, which are barely observable at room temperature in solution, are clearly seen at 4.0 K (Inset of Figure 2a). They can be assigned to the spin-forbidden ^4^A_2_ → ^2^T_2_ transition.^19^

**Figure 2:**
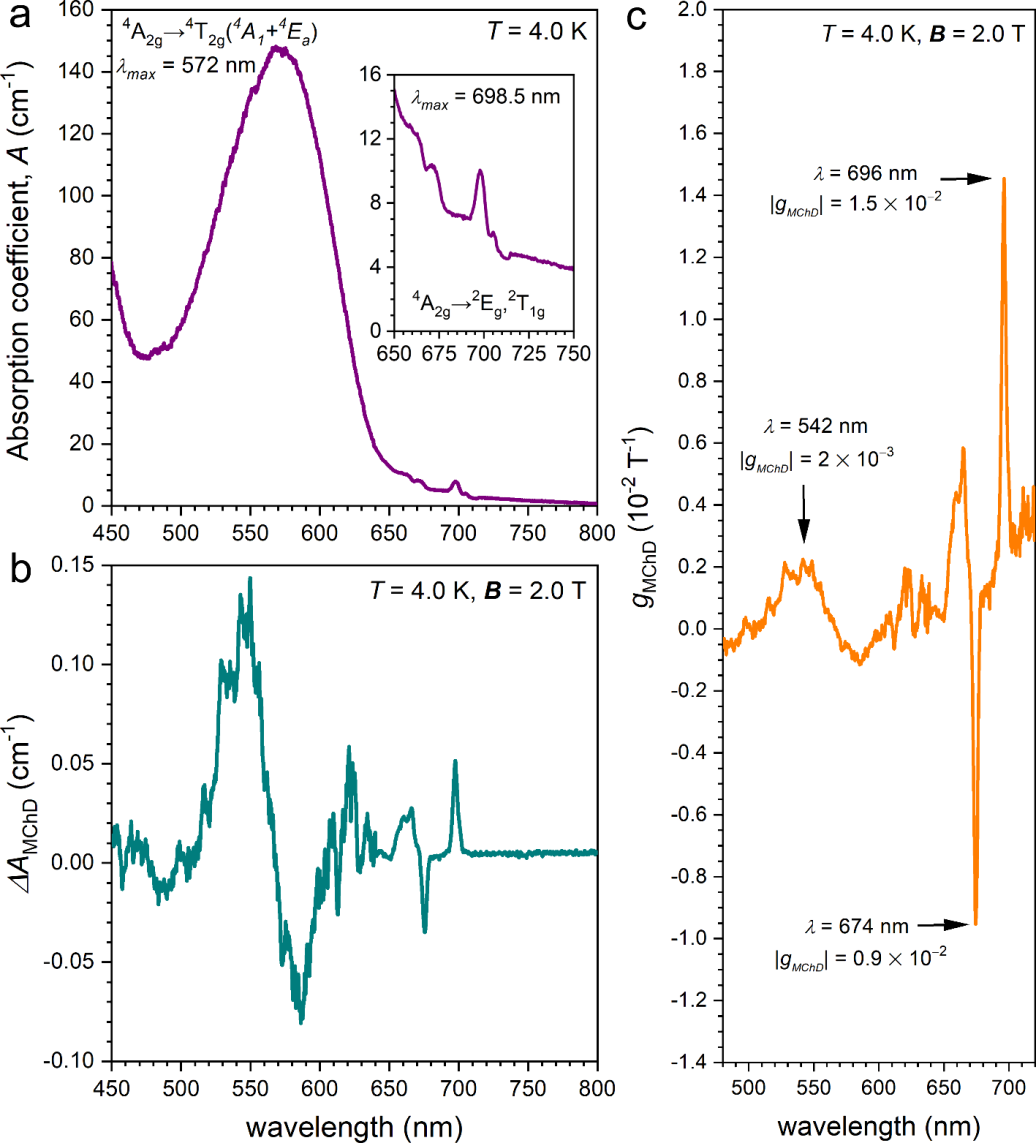
Absorption (a) and *ΔA*_MChD_ (b) spectra of (*Λ*)-**1** dispersed in a KBr pellet at *T* = 4.0 K and ***B*** = 2.0 T. Inset of panel a shows the detail of the absorption between 650 and 750 nm. *g*_MChD_ plot in the 480–720 nm range (c) highlighting the most MChD-active electronic transitions and the associated *g*_MChD_ values.

The MChD spectrum at *T* = 4.0 K and ***B*** = 2.0 T obtained as the difference between the light absorption under a magnetic field ***B*** applied parallel (***B*** ↑↑ ***k***) and antiparallel (***B*** ↓↑ ***k***) with respect to the unpolarized light wavevector ***k*** is reported in Figure 2b. Two sharp absorptive-shape contributions of similar intensity and opposite sign associated with the spin-forbidden ^4^A_2_ → ^2^T_2_ and ^4^A_2_ → ^2^T_1_, ^2^E transitions are observed at λ = 674 and 696 nm, respectively. A broader dispersive-shape contribution associated with the spin-allowed ^4^A_2_ → ^4^T_2_ transition is observed between 500 and 620 nm. The relative intensity of the MChD signals with respect to the absorption at zero field, that is the *g*_MChD_ dissymmetry factor (Equation S3), is shown in Figure 2c as a function of the irradiation wavelength. The two strongest contributions, of the order of 1 × 10^–2^ T^–1^, are associated with the spin-forbidden transitions, while that associated with the spin-allowed transition is one order of magnitude lower.

Variable temperature (4.0–150 K) and variable magnetic field (0–2.0 T) MChD investigations were done to obtain better insights into the response of the MChD intense spin-forbidden transitions (Figure 3). The magnetic field variation shows a signal that increases linearly up to 2.0 T (Figure 3a) as expected for a system featuring paramagnetic noninteracting ions. The thermal dependence shows MChD signals that lose intensity as the temperature increases with a linear dependence with 1/*T* (Figure 3b). As the temperature increases, the shape of the signal changes from a pure absorptive-shape at 4.0 K to a dispersive-shape at 150 K. This clearly indicates that at low temperature, the origin of the MChD signals is due to a difference in Boltzmann population of the ground state split by the magnetic field (MChD *C*-term). The MChD *C*-term, as for the Faraday *C*-term in MCD spectroscopy, is temperature dependent (∝ 1/*T*) and has an absorptive line shape.^25,26^ The dispersive line shape of the signal at *T* = 150 K is instead indicative that the differential absorption arises from the temperature-independent Zeeman splitting of the ground and excited states induced by the magnetic field, e.g., a MChD *A*-term, which is the analogous to the Faraday *A*-term in MCD spectroscopy.^25,26^ At 290 K, the intensity of the MChD signal is below the detection limits of our experimental setup. However, the signal shape at *T* = 150 K, being already dominated by the MChD *A*-term, can be used as a reference MChD spectrum for room temperature experiments (see below). The room temperature MChD spectrum of enantiopure (*Λ*)-**1** and (*Δ*)-**1** in solution was already reported,^16^ and its shape and sign are in agreement with the results reported herein.

**Figure 3:**
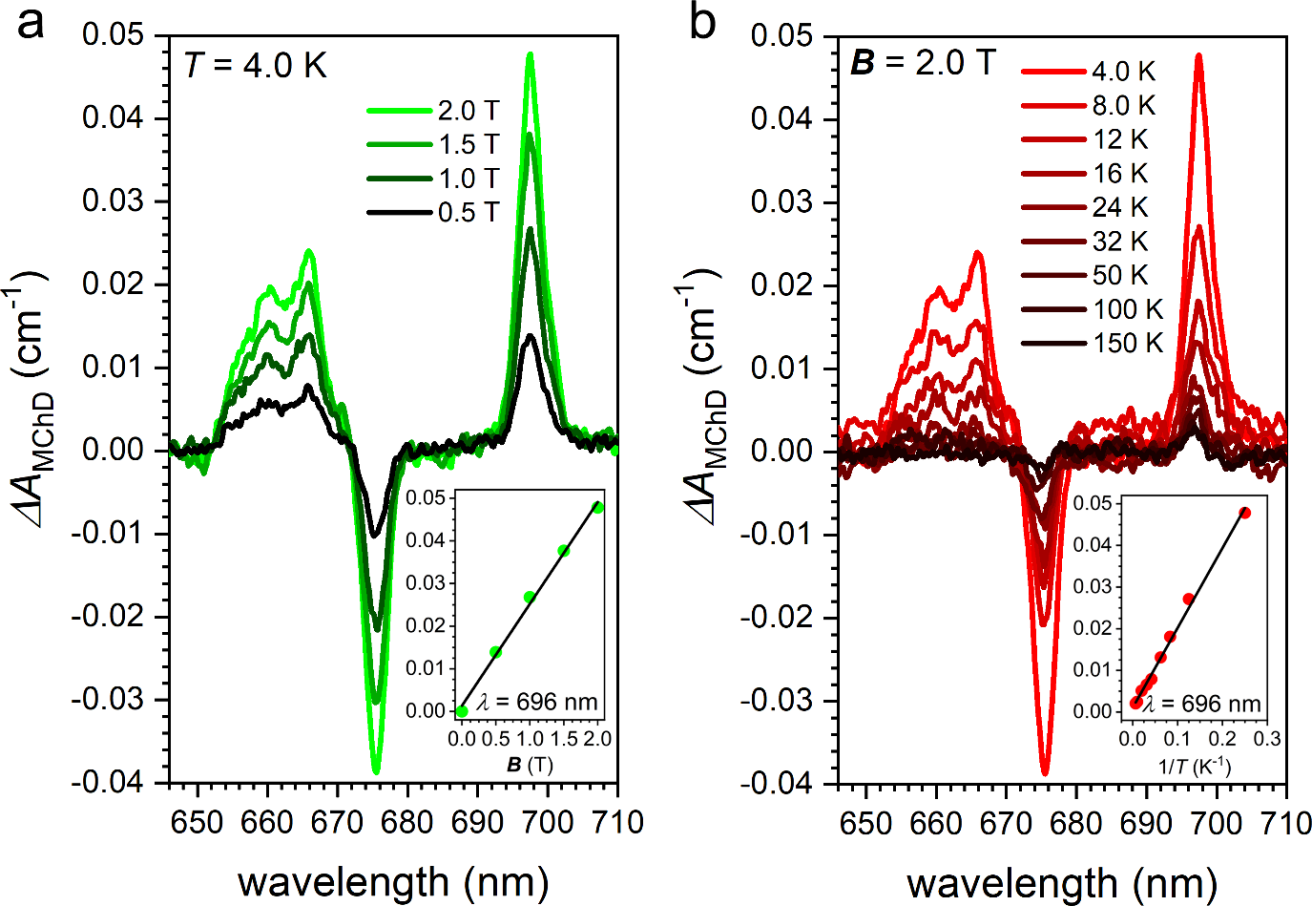
Magnetic field (a) and temperature (b) dependence of the *ΔA*_MChD_ signal for (*Λ*)-**1** dispersed in a KBr pellet. Insets show the linear dependence of *ΔA*_MChD_ over ***B*** and 1/*T* in the investigated ranges (0.0–2.0 T and 4.0–150 K) at λ = 696 nm.

Magneto-chiral photochemistry (MChPh) experiments were performed irradiating a 0.03 M aqueous solution of (*rac*)-**1** at *T* = 5 °C placed in a standard 1 × 1 cm^2^ UV–vis quartz cuvette with a depolarized titanium:sapphire continuous wave laser source able to provide irradiation powers up to 500 mW within the 690–710 nm range with emission line widths of ca. 40 GHz.

Given the relationship between the MChD response and MChPh efficiency, the irradiation wavelength corresponding to the *g*_MChD_ maximum (λ = 695.5 nm) was used to investigate the irradiation time dependence of the induced *ee*. Experiments lasting 15, 30, and 45 min were performed to determine the optimal duration of the experiments described below. A NCD spectrum showing the same spectral shape and sign of the enantiopure (*Λ*)-**1** (Figure 1), but with lower *Δε*_NCD_ intensities, was clearly observed after 15 min of irradiation (Figure S6). This shows that enantioselective MChPh is taking place, and it is easily detectable using standard NCD spectroscopy. Indeed, the *ee* was detected by rapidly transferring the sample cuvette into a commercial NCD spectrometer and collecting the NCD spectra within a wide spectral range (350–800 nm) 2 min after the interruption of the laser irradiation (see experimental section for details). This approach, compared to previous single-wavelength detection methods,^15^ provides a full NCD spectrum that unambiguously shows that the induced *ee* corresponds to tris­(oxalato)­chromate­(III) rather than any other chiral degradation products. After 30 min of irradiation, the *ee* starts to saturate and reaches 94% of the *ee* value obtained at 45 min irradiation (Figure S6). Therefore, 30 min was chosen as the reference irradiation time for all following experiments.

Wavelength-dependent studies within the 692.5–698.5 nm range were performed to define the irradiation wavelength that provides the highest *ee*. The results are reported in Figure 4a, while in Figure 4b the wavelength dependence of the *Δε*_NCD_ maximum at λ = 552 nm is compared to the MChD spectrum obtained at *T* = 150 K.

**Figure 4:**
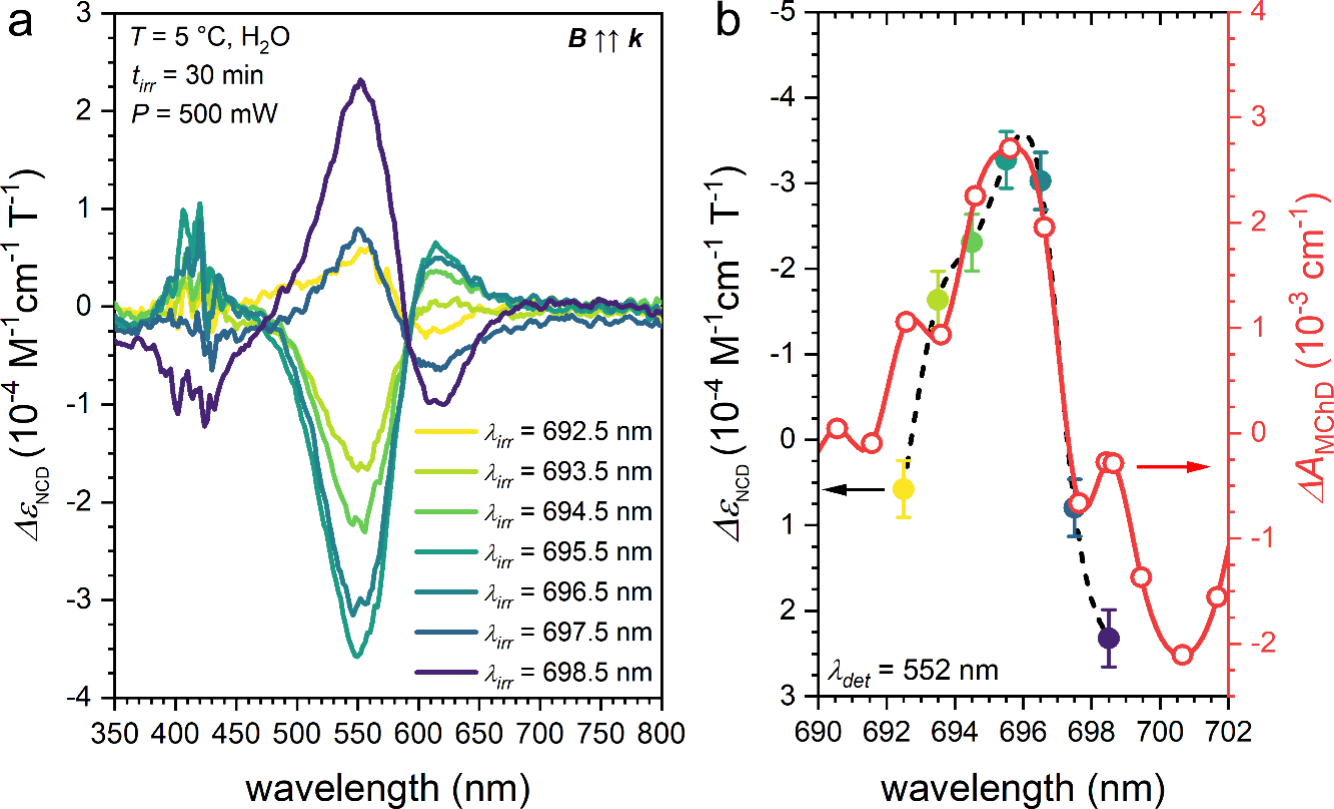
(a) NCD spectra corresponding to the induced *ee* through MChPh as a function of the irradiation wavelength (see the legend) and (b) wavelength dependence of the *Δε*_NCD_ (λ = 552 nm) compared to the *ΔA*_MChD_ signal at *T* = 150 K in the same spectral region.

It can be clearly seen that tuning the irradiation wavelength from λ = 692.5 to 695.5 nm, the induced *ee* increases, and then it decreases to almost zero at λ = 697.5 nm and then increases again at λ = 698.5 nm but with an opposite sign. The wavelength dependence obtained by MChPh is in very good agreement with the MChD spectral profile (Figure 4b), which indicates that MChD is at the origin of the induced *ee*. Comparing the sign of the induced *ee* with that of the enantiopure forms, it can be highlighted that when the laser irradiation wavevector ***k*** is antiparallel with respect to the magnetic field pseudovector ***B*** it provides an *ee* of (*Δ*)-**1** when irradiating at λ = 695.5 nm and an *ee* of (*Λ*)-**1** when irradiating at λ = 698.5 nm. The change in sign of the induced *ee* as a function of the irradiation wavelength and a maximum of *ee* at λ = 695.5 nm are both in agreement with the seminal work of Rikken and Raupach.^15^

The irradiation wavelength that provides the highest *ee*, λ_irr_ = 695.5 nm, was selected to perform experiments as a function of the applied magnetic field. Magnetic fields ***B*** up to 30 T applied parallel and antiparallel with respect to the laser beam ***k*** were used to investigate MChPh at *T* = 5.0 and 18.0 °C. The ***B*** dependence of the induced *ee* is reported in Figure 5 together with the NCD spectra obtained for the experiments at *T* = 5.0 °C. The NCD spectra for the experiments at *T* = 18 °C are reported in Figure S7.

**Figure 5:**
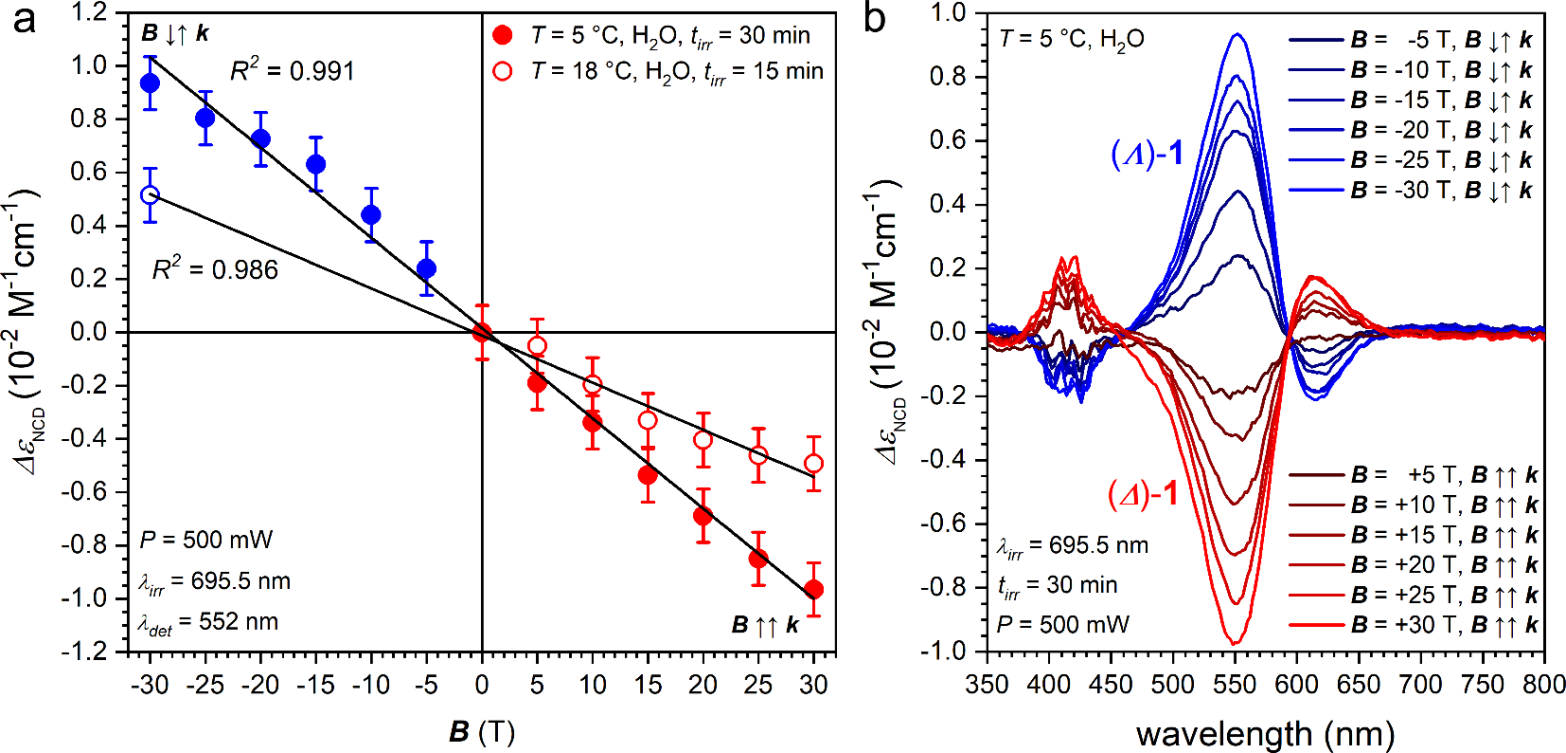
(a) Magnetic field dependence of the induced *Δε*_NCD_ (λ = 552 nm) through MChPh experiments performed at *T* = 5.0 and 18 °C (see legend) at magnetic fields ***B*** up to 30 T parallel and antiparallel applied with respect to the laser beam *k* (λ = 695.5 nm). (b) Wide range (350–800 nm) NCD spectra obtained for each experiment (see legend).

When ***B*** ↑↑ ***k***, an *ee* of (*Δ*)-**1** that increases linearly up to 30 T is obtained. The *ee* is smaller at 18 °C with respect to 5 °C due to the faster racemization process.^27^ When ***B*** ↓↑ ***k***, an *ee* equal in magnitude but of the opposite enantiomer, (*Λ*)-**1**, is obtained. The variation of the enantioenriched enantiomer as a function of the relative orientation of magnetic field ***B*** and ***k*** orientation is a further proof that MChD is driving the photochemistry.^15^ The linearity of *ee* versus ***B*** shows that much higher *ee* can be obtained with higher fields.

A calibration curve prepared from pure enantiomers obtained by chemical resolution (see experimental section for details) allows us to quantitatively determine the induced *ee* (Figure S8). The induced *Δε*_NCD_ at *T* = 5 °C and ***B*** = 30 T corresponds to an *ee* of 0.50(3)%. This value compares well with the typical *ee* obtained by CPPh (see below).

The racemization rate of the induced *ee* was also studied. NCD spectra collected as a function of the time after MChPh experiments under ***B*** = 30 T show an exponential decay with a half-time of 35(1) min (Figure S9). This value compares well with that of the enantiopure (*Λ*)-**1** obtained by chemical resolution (Figure S10) and literature findings,^24^ and it further proves that the generated *ee* is associated with tris­(oxalato)­chromate­(III).

MChPh experiments were also performed by irradiating at λ = 532 nm, a wavelength close to the maximum NCD response of the spin-allowed ^4^A_2_ → ^4^E_*a*_ transition (λ = 552 nm, see above), by means of a frequency doubled Nd:YAG laser. The detected *Δε*_NCD_ (λ = 552 nm) corresponds to an *ee* of 0.002%, that is two orders of magnitude lower that that obtained irradiating at λ = 698.5 nm in the same experimental conditions (Figure S11a). This result is in agreement with the difference in the *g*_MChD_ values for the two electronic transitions obtained through MChD measurements. Indeed, the MChD response for the spin-allowed transition is weak even if the NCD response is high. Therefore, to compare the efficiency of MChPh and CPPh in generating *ee*, time-dependent CPPh experiments with right (*R*) and left (*L*) CPL irradiation at λ = 532 nm in the absence of a magnetic field were performed.

A NCD spectrum showing the same spectral shape and sign of the enantiopure (*Λ*)-**1**, but with lower *Δε*_NCD_ intensities, was clearly observed after 5 min of irradiation with *R*-CPL (Figure S11b). Longer experiments provide a higher *ee*, reaching saturation after 30 min of laser irradiation. Irradiation with *L*-CPL provides an *ee* of the opposite enantiomer, (*Δ*)-**1**, which confirms that a partial photoresolution of **1** is taking place (Figure S11b). The results are in agreement with similar studies performed in the past under different experimental conditions.^27^ The highest detected |*Δε*_NCD_| (λ = 552 nm) is ca. 4.5 × 10^–2^ M^–1^ cm^–1^, which is three orders of magnitude higher than that obtained irradiating with unpolarized light in a magnetic field (***B*** = 30 T) at λ = 532 nm (Figure S11a and b). The *Δε*_NCD_ value corresponds to an *ee* of 1.66(1)%.

Finally, CPPh experiments were performed with *R*- and *L*-CPL irradiation at λ = 698.5 nm, the wavelength that provided the maximum *ee* through MChPh. The highest detected |*Δε*_NCD_| is ca. 2.4 × 10^–3^ M^–1^ cm^–1^, which is one order of magnitude smaller than that obtained irradiating at λ = 532 nm (Figure S11). The corresponding *ee* can be estimated to be ca. 0.12%, which is lower than that obtained by MChPh.

These results demonstrate that by a careful choice of the irradiation wavelength, hence of the involved electronic transition, MChPh can provide an *ee* of the same order of magnitude of CPPh. The high room-temperature *g*_NCD_ value for the ^4^A_2_ → ^4^E is indeed responsible for the high *ee* observed irradiating at λ = 532 nm by CPPh, whereas the *g*_MChD_ value associated with the ^4^A_2_ → ^2^T_1_, ^2^E drives the *ee* obtained by MChPh at λ = 695.5 nm.

It should be highlighted that our CPPh experiments have been performed with a degree of circular polarization >95% and cannot yield a higher *ee*. On the contrary, the linear response of MChPh with the intensity of the applied magnetic field ***B*** shows that a higher *ee* should be obtained under higher magnetic fields. Another strategy would consist of choosing chiral metal complexes with higher *g*_MChD_ factors than tris­(oxalato)­chromate­(III) to yield a higher *ee* at the same field intensity and irradiation power used in this study.

### Conclusion and Perspectives

In conclusion, we have investigated the MChPh of tris­(oxalato)­chromate­(III) in water solution at different temperatures and with magnetic fields of up to 30 T. A new experimental protocol, based on *i)* the detection of the induced *ee* collecting the NCD spectrum of the irradiated solution in a wide wavelength range and *ii)* a calibration curve prepared from pure enantiomers obtained by chemical resolution, allows one to quantitatively determine the induced *ee* without ambiguity.

An *ee* of ca. 0.50% has been achieved by irradiating (*rac*)-**1** at *T* = 5 °C for 30 min at λ = 695.5 nm (500 mW) under a magnetic field ***B*** = 30 T. By changing the relative orientation of ***B*** and ***k***, an *ee* of the same magnitude but opposite sign was obtained as predicted by the MChD theory. Wavelength-dependent MChD and MChPh studies showed that the obtained *ee* by MChPh is intimately related in magnitude and sign to the MChD of the chromophore. Accordingly, our recent efforts to understand the physicochemical parameters that provide the highest MChD responses in enantiopure chiral molecular systems^28–37^ form a solid basis to identify those systems that can provide the highest *ee* through MChPh.

Magnetic field dependent studies up to 30 T showed that the induced *ee* varies linearly with the magnetic field without any sign of saturation owing to the Zeeman splitting at the origin of the corresponding MChD signal at *T* > 150 K. Systems featuring fast or ultrafast photochemical reactivity might be suited for experiments under pulsed magnetic fields (up to 100 T) to generate a much higher *ee*.

Finally, these experiments and the perspectives they open up demonstrate that, for systems involving (transient) paramagnetic species, MChPh can definitely compete with CPPh in generating a sizable *ee*, reinforcing the relevance of MChPh as one of the potential mechanisms at the origin of molecular homochirality.

## Supplementary Material





## Notes

Experimental section and additional spectroscopic data (PDF)
Transparent Peer Review report available (PDF)
