# Supplementary material for: Enantioselective Magneto-Chiral Photochemistry Rediscovered
Source: ACS Cent Sci. 2025 Jun 16;11(7):1147–53. doi: 10.1021/acscentsci.5c00772 (PMC12291129; doi:10.1021/acscentsci.5c00772)
Supplement: Supplementary file 1 [file oc5c00772_si_001.pdf]

Enantioselective Magneto-Chiral Photochemistry Rediscovered

Maria Sara Raju, Maxime Aragon-Alberti, Kevin Cardenas, Ivan Breslavetz, Geert L. J. A. Rikken, Cyrille Train, and Matteo Atzori*

Laboratoire National des Champs Magnétiques Intenses (LNCMI), CNRS, Univ. Grenoble Alpes, INSA Toulouse, Univ. Toulouse Paul Sabatier, EMFL, F-38042 Grenoble – France.

Corresponding author:

Dr. Matteo Atzori, matteo.atzori@lncmi.cnrs.fr

SUPPLEMENTARY INFORMATION

EXPERIMENTAL SECTION

Synthesis. (Δ)-$K_2[Sb_2(C_2H_2O_6)]\cdot 3H_2O$, (*rac*)-$[Ni(1,10\text{-phen})_3]Cl_2$, ($\Lambda$)-$[Ni(1,10\text{-phen})_3](ClO_4)_2$, ($\Delta$)-$[Ni(1,10\text{-phen})_3](ClO_4)_2$, (*rac*)-$K_3[Cr(C_2O_4)_3]\cdot 3H_2O$, ($\Delta$)-$K_3[Cr(C_2O_4)_3]\cdot 3H_2O$ and (Λ)-$K_3[Cr(C_2O_4)_3]\cdot 3H_2O$ were prepared following the published procedures.[17-20]

UV-vis absorption spectroscopy in solution. Absorption spectra in solution (350-800 nm) were collected on 0.03 M solution of **1** in H_2O, H_2O:DMSO (1:1) or DMSO placed in quartz suprasil QS cuvettes with 1 cm optical path using a double-beam JASCO V-770 absorption spectrophotometer.

Natural Circular Dichroism Spectroscopy. Natural Circular Dichroism spectra in solution (350-800 nm) were collected on 0.03 M solution of (Δ)-**1** and (Δ)-**1** in H_2O placed in quartz suprasil QS cuvettes with 1 cm optical path using a JASCO J-1500 circular dichroism spectrophotometer.

The NCD dissymmetry factor g_{NCD} is defined as follows:

$$g_{NCD} = \frac{\Delta\varepsilon_{NCD}}{\varepsilon} \qquad \text{(eq. S1)}$$

where $\Delta\varepsilon_{NCD}$ is the differential absorption coefficient between left- and right-circularly polarized light and ε is the absorption coefficient of the system measured with unpolarized light.

The calibration curve was prepared as follows: an initial 0.03 M solution of enantiopure (Λ)-**1** was prepared by dissolving 73 mg of (Λ)-**1** in 5 mL of 1:1 by volume mixture of DMSO:H_2O. The NCD spectrum was collected immediately after the preparation of the solution to avoid thermal racemization. This mixture of solvent was chosen because addition of DMSO to H_2O strongly reduces the racemization rate of **1**.[16] A portion of this solution was transferred into a volumetric flask and diluted with a 0.03 M DMSO:H_2O (1:1) solution of (*rac*)-**1** to keep the total concentration of **1** constant. The same procedure is repeated to prepare further diluted solutions. An NCD spectra was collected for each solution and the variation of intensity of the peaks were plotted as a function of the enantiomeric excess to obtain the calibration curve.

Magnetic Circular Dichroism Spectroscopy. Magnetic Circular Dichroism spectra in solution (350-800 nm) were collected on 0.03 M solution of **1** in H_2O placed in quartz suprasil QS cuvettes with 0.5 cm optical path using a JASCO J-1500 circular dichroism spectrophotometer equipped with 1.6 T permanent magnet. Measurements were done with the magnetic field polarity applied parallel and antiparallel with respect to the light beam. The half-difference between the spectra recorded with $\boldsymbol{B}\uparrow\uparrow\boldsymbol{k}$ and $\boldsymbol{B}\downarrow\uparrow\boldsymbol{k}$ provides the $\Delta\varepsilon_{MCD}$ spectra.

The MCD dissymmetry factors g_{MCD} is defined as follows:

$$g_{MCD} = \frac{\Delta\varepsilon_{MCD}}{\varepsilon\,\boldsymbol{B}} \qquad \text{(eq. S2)}$$

where $\Delta\varepsilon_{MCD}$ is the differential absorption coefficient between left- and right-circularly polarized light propagating along a magnetic field of intensity $\boldsymbol{B}$, and ε is the absorption coefficient of the system measured with unpolarized light at zero field.

Magneto-Chiral Dichroism Spectroscopy. Magneto-Chiral Dichroism spectra were recorded with a home-made multichannel MChD spectrometer operating in the visible and near infrared spectral window (400−1600 nm) between 3.0 and 290 K with an alternating magnetic field $\boldsymbol{B}$ up to ±2.0 T with a fast-sweeping superconducting magnet. MChD spectra were acquired on (*Λ*)-**1** dispersed on KBr pellets. The spin-allowed and spin-forbidden electronic transitions had to be probed in KBr pellets with different concentrations to optimize the detection limits of the MChD signals for the weak absorption peak centered at λ = 698.5 nm. Thus, the spin allowed transition was probed on a pellet half as concentrated (16.5 wt %) as the pellet used to probe spin-forbidden transitions (34.59 wt %). The samples were mounted on a titanium sample holder over a 0.9 mm hole diameter centered with respect to a 1.0 mm diameter collimated beam. Measurements were performed in the 4.0−150 K range with an alternating magnetic field $\boldsymbol{B}$ = ±2.0 T and frequency Ω = 0.04 Hz. MChD spectra as a function of the magnetic field were recorded at T = 4.0 K for alternating magnetic fields of different amplitudes (0.0-2.0 T). Unpolarized light was provided by a broadband Energetiq – Hamamatsu Laser Driven Light Sources (EQ-99X-FC-S or EQ-77X-FC-S). MChD spectra were obtained at each temperature/magnetic field value by collecting, on average, 50.000 spectra, with an integration time of 50 ms. The spectra were collected with a high resolution/high sensitivity Optosky detector equipped with a thermoelectric cooled sensor operating in the 200–1000 nm spectral region with an analogic/digital convertor of 16 bits. Each spectrum was correlated to a specific magnetic field value by a dual channel digitizer (Picoscope 5000B) acquiring simultaneously triggers from the spectrometer and the magnetic field from a calibrated Hall effect sensor (Lakeshore) placed in proximity of the sample. Data were then post-processed as a synchronous detection with a specific MatLab routine to obtain the MChD spectra. ΔA_{MChD} values were corrected by the sample thickness and concentration.

The MChD dissymmetry factor g_{MChD} is defined as follows:

$$g_{MChD} = \frac{\Delta A_{MChD}}{A\,\boldsymbol{B}} = \frac{(A(\boldsymbol{B}\uparrow\uparrow\boldsymbol{k}) - (A(\boldsymbol{B}\downarrow\uparrow\boldsymbol{k}))}{A\,\boldsymbol{B}} \qquad \text{(eq. S3)}$$

where ΔA_{MChD} is the differential absorption coefficient between the light absorption collected under a magnetic field parallel and antiparallel oriented with respect to the light wavevector $\boldsymbol{k}$, A is the effective absorption coefficients of the electronic transitions at zero field and $\boldsymbol{B}$ is the applied magnetic field intensity.

Magneto-Chiral Photochemistry Experiments. The experiments were done on 0.03 M H_2O solutions of **1** placed in a quartz suprasil QS cuvette with 1 cm optical path actively thermostated at T = 5 °C or 18 °C during the experiments with a *LAUDA* thermostatic bath and before the experiment with a standard fridge. The applied magnetic fields in the $\boldsymbol{B}$ = ±30 T (bore diameter 50 mm) were provided by the resistive magnets of the LNCMI-CNRS which is part of the European Magnetic Field Laboratory. The laser beam was generated by a Titanium:Sapphire continuous wave laser source (3900S Millennia) able to provide irradiation powers up to 500 mW within the 680-710 nm range with linewidths of ca. 40 GHz. The laser was depolarized using two cascaded photoelastic modulators plus two liquid-crystal based depolarizers and passing the light into 6 m of a 100 μm diameter optical fiber before reaching the solution placed in the center of the magnet. NCD spectra were collected before each experiment to subtract the background NCD signal due to a weak linear dichroism of the cuvette. NCD spectra were collected after each experiment 2 minutes

after the laser beam irradiation was switched off moving the cuvette into the JASCO J-1500 spectrophotometer. Racemization studies were done acquiring NCD spectra continuously for 70 and 220 minutes for the pure enantiomer and the enantioenriched solution respectively. Photochemistry experiments under CPL irradiation were done in similar experimental conditions without depolarizing the laser beam but generating *R* and *L*-CPL interposing first a $\lambda/2$ waveplate and then a $\lambda/4$ waveplate (Thorlabs) between the laser source and the cuvette, working in free-beam conditions.

ADDITIONAL FIGURES

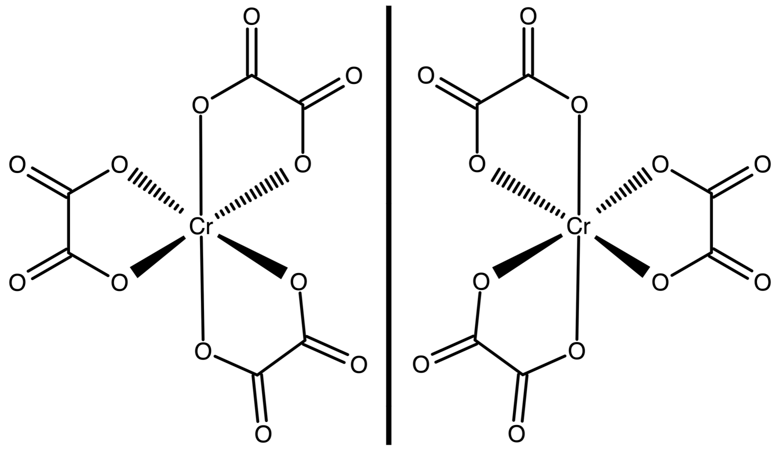

Figure S1. Molecular structure of the Δ (left) and Λ (right) tris(oxalate)chromate(III) complex anions.

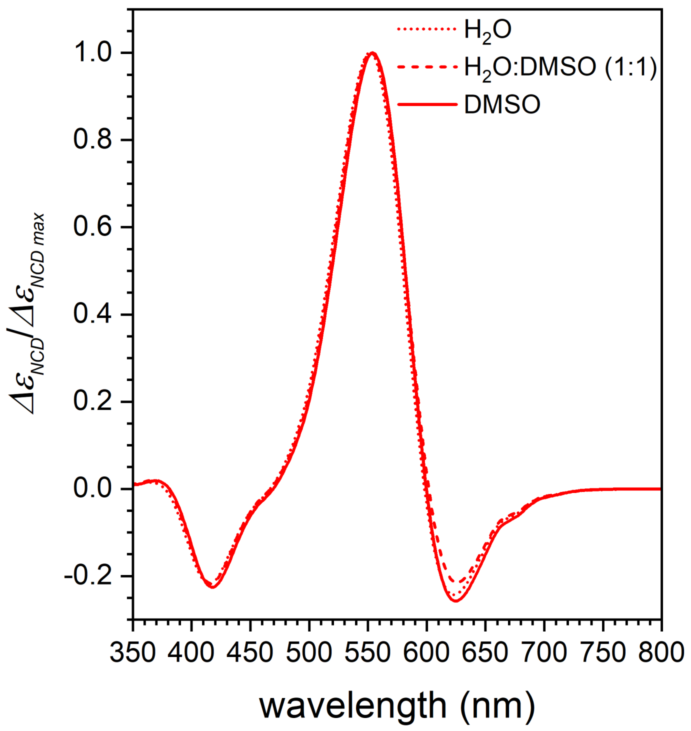

Figure S2. Room temperature normalized absorption NCD spectrum (350-800 nm) of enantiopure (Λ)-**1** in three different solvents (see legend).

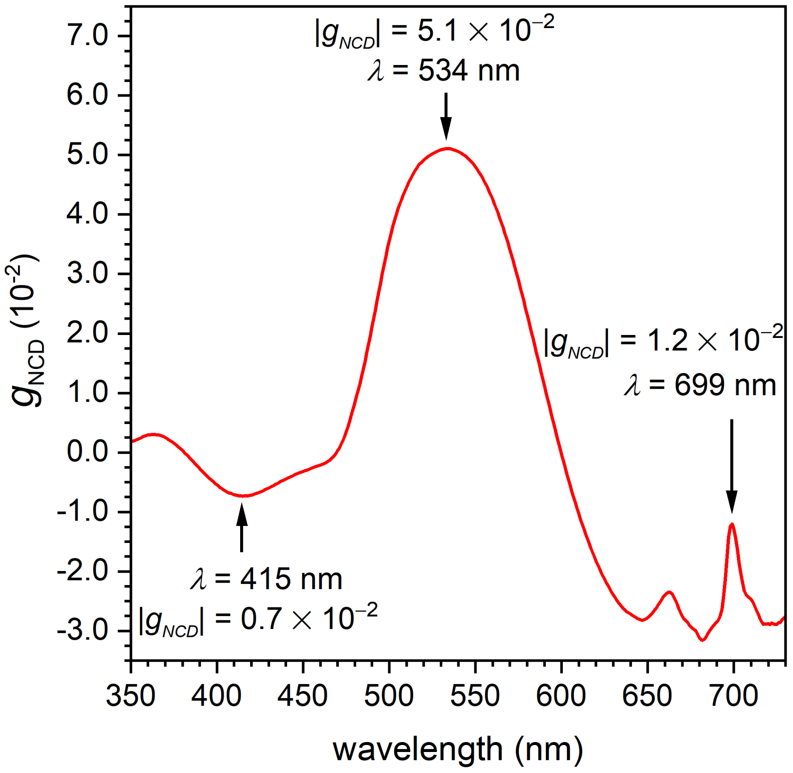

Figure S3. Plot of the g_{NCD} dissymmetry factor at room temperature for (*Λ*)-**1** in DMSO solution.

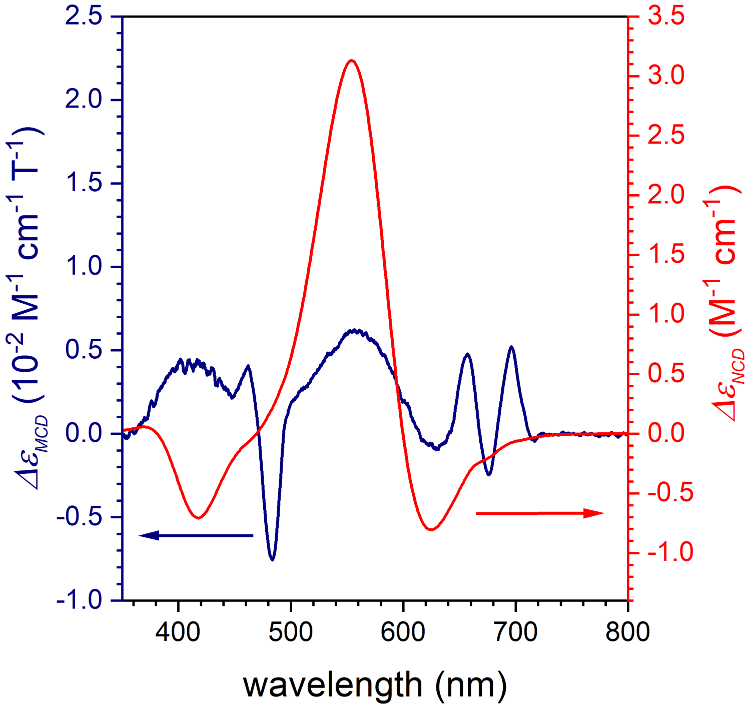

Figure S4. Room temperature Magnetic-Circular Dichroism (MCD) spectrum (350-800 nm) of **1** obtained under a magnetic field ***B*** = 1.6 T compared to the NCD spectrum of (*Λ*)-**1** in DMSO solution.

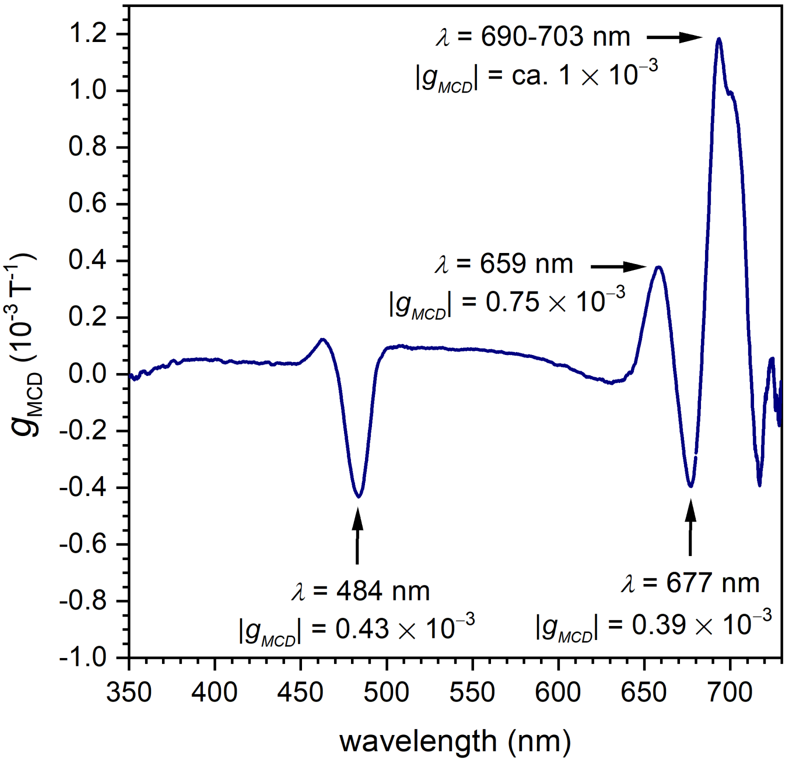

Figure S5. Plot of the g_{MCD} dissymmetry factor at room temperature for **1** in DMSO solution.

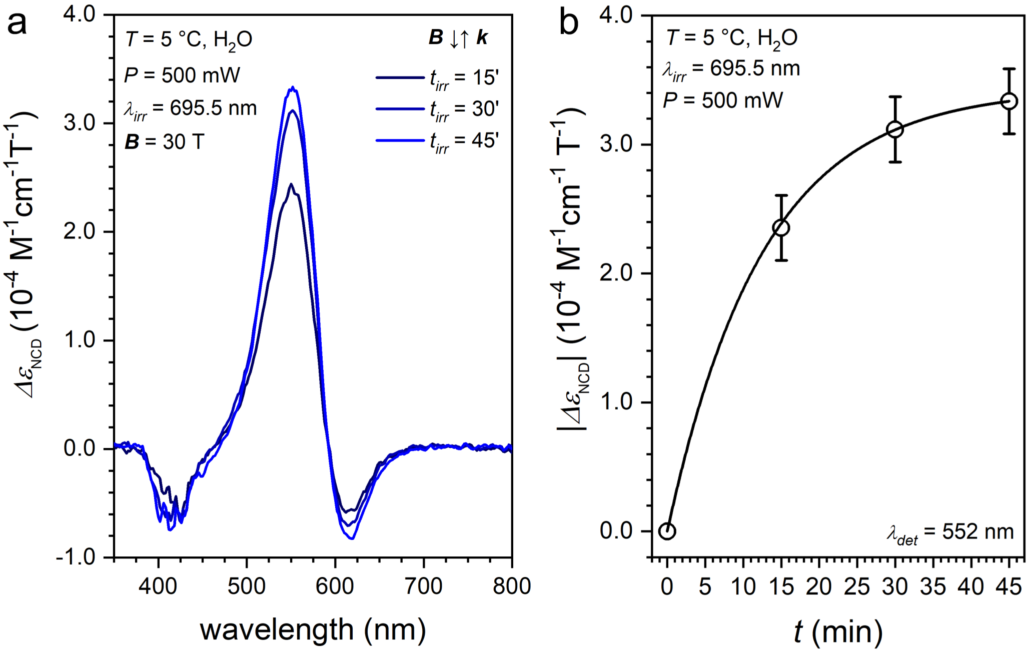

Figure S6. Wide-range NCD spectra (350-800) corresponding to the induced *ee* obtained through MChPh experiments on **1** (see legend for details) as a function of the irradiation time (a) and plot of the time variation of the NCD maximum at $\lambda = 552$ nm (b).

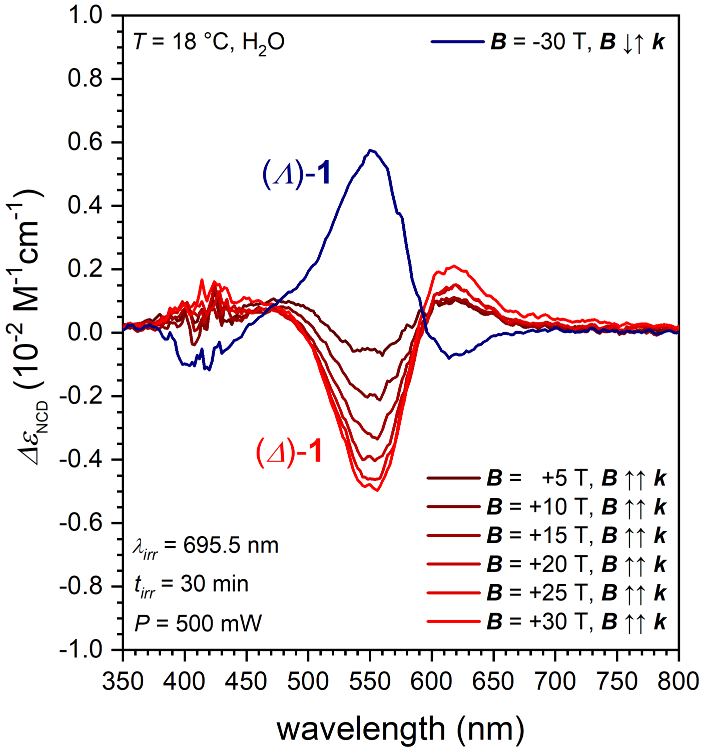

Figure S7. Wide-range NCD spectra (350-800) corresponding to the induced *ee* obtained through MChPh experiments on **1** at $T = 18$ °C as a function of the magnetic field intensity.

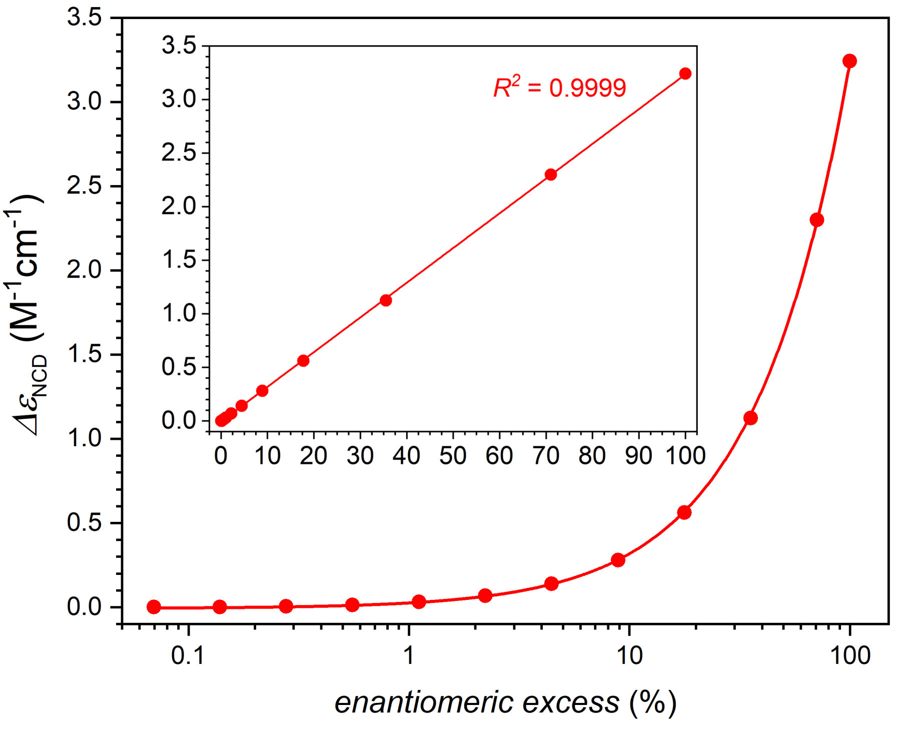

Figure S8. Calibration curve used to determine the *ee* on the basis of the $\Delta\varepsilon_{NCD}$ maxima at $\lambda = 552$ nm.

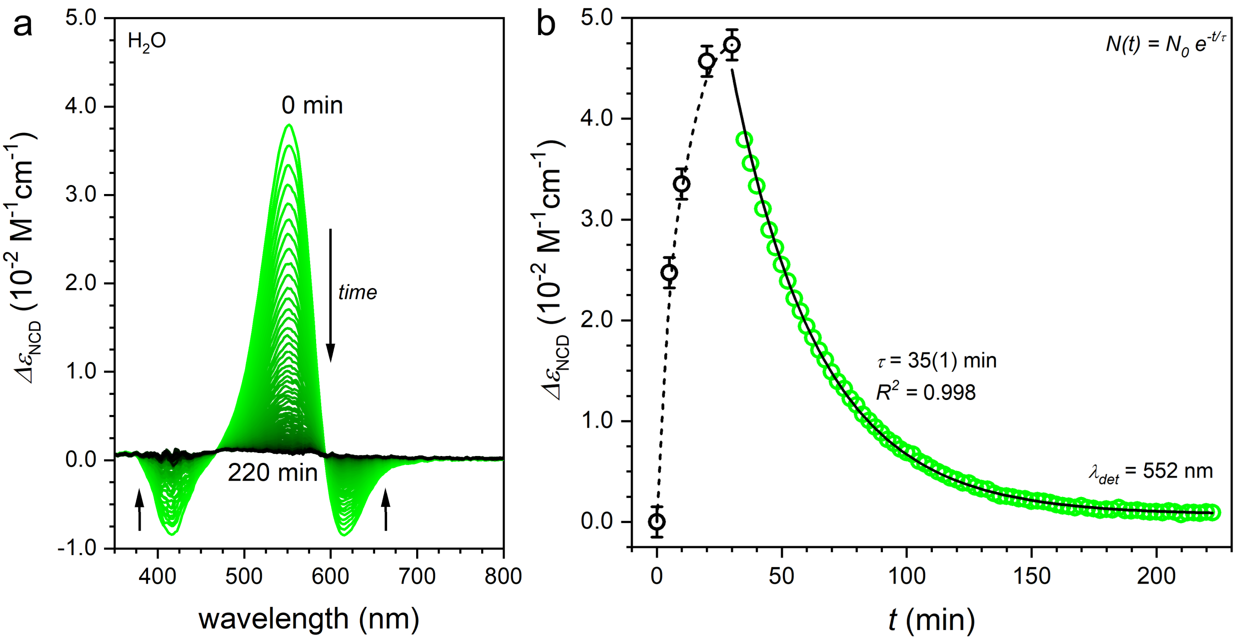

Figure S9. Temporal decay of the induced *ee* after MChPh experiments on **1** at T = 5 °C and $\boldsymbol{B}$ = 30 T in H_2O solution detected through wide range NCD spectroscopy (350-800) nm (a) and fitting of the data ($\Delta\varepsilon_{NCD}$ at λ = 552 nm) to extract the half-time of the racemization process.

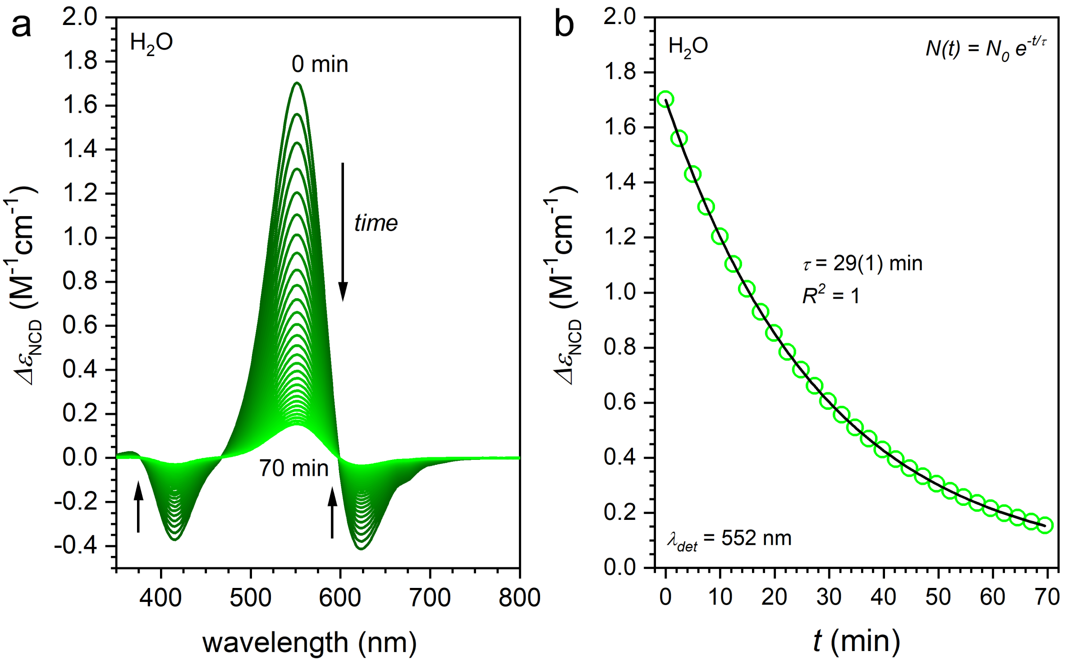

Figure S10. Temporal decay of the enantiopure (Λ)-**1** in H_2O solution detected through wide range NCD spectroscopy (350-800) nm (a) and fitting of the data ($\Delta\varepsilon_{NCD}$ at λ = 552 nm) to extract the half-time of the racemization process.

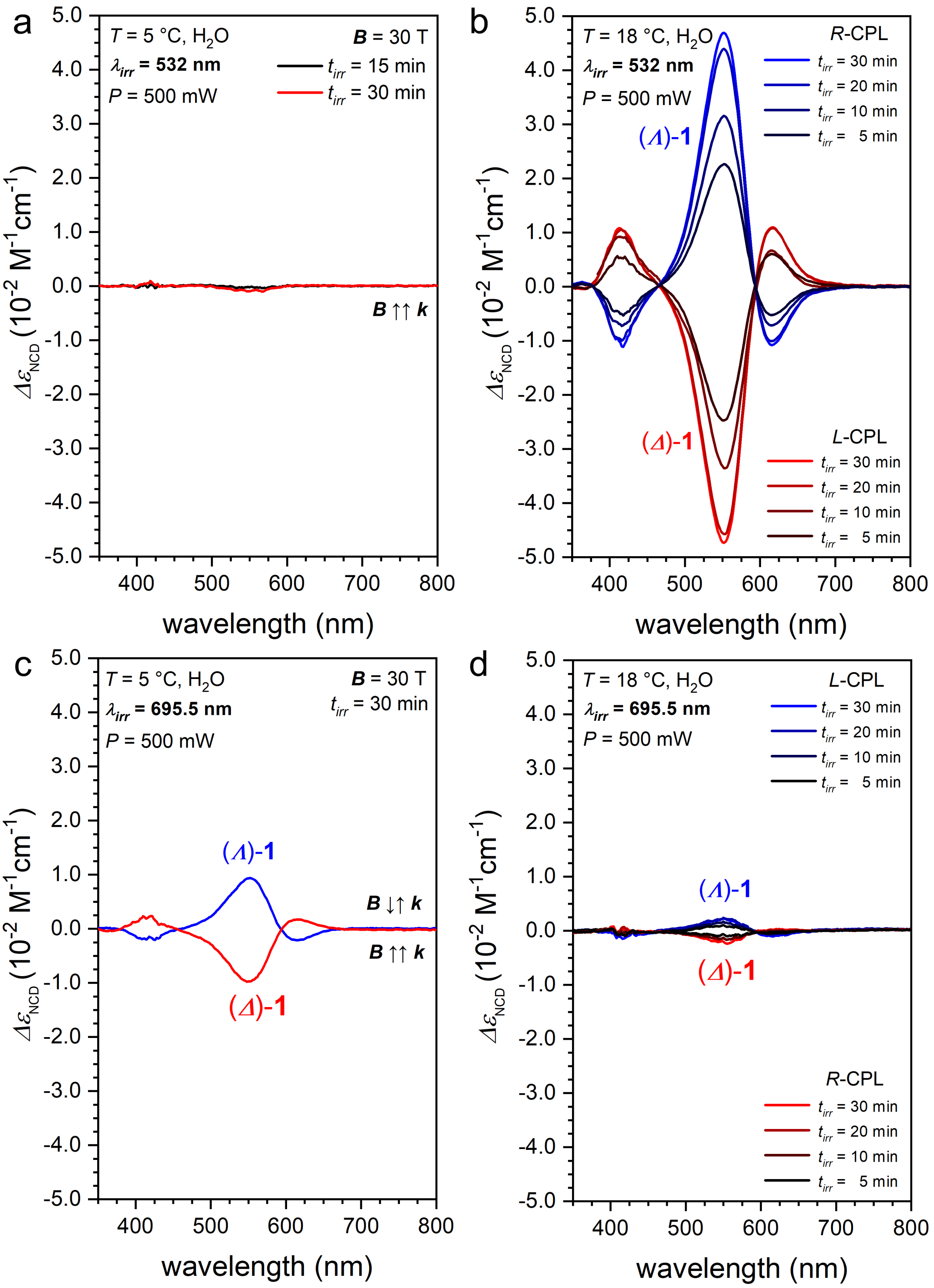

Figure S11. Wide-range NCD spectra (350-800) corresponding to the induced *ee* obtained through MChPh (a) and CPPh (b) on **1** at λ_{irr} = 532 nm, and MChPh (c) and CPPh (d) on **1** at λ_{irr} = 695.5 nm.
