# Supplementary material for: Enantioselective Magneto-Chiral Photochemistry Rediscovered
Source: ACS Cent Sci. 2025 Jun 16;11(7):1147–53. doi: 10.1021/acscentsci.5c00772 (PMC12291129; doi:10.1021/acscentsci.5c00772)
Supplement: Supplementary file 2 [file oc5c00772_si_002.pdf]

oc-2025-00772f.R1

Name: Peer Review Information for "Enantioselective Magneto-Chiral Photochemistry Rediscovered"

First Round of Reviewer Comments

Reviewer: 1

Comments to the Author

Magneto-chiral phenomena, which involve a subtle interplay of chirality and magnetism involving unpolarized light propagating collinear with a static magnetic field, are attracting increasing interest both as a new form of chiroptical spectroscopy and for novel technological applications. However, one important aspect, namely magneto-chiral photochemistry for generating an enantiomeric excess, has languished since its first demonstration 25 years ago by one of the authors. Inter alia, this has fundamental relevance for the origin of biomolecular homochirality since it depends on unpolarized light and static magnetic fields which are far more ubiquitous in the cosmos than the circularly polarized light upon which circularly polarized photochemistry (CPPh), currently the most accredited mechanism, depends.

This paper revisits the original MChPh photoresolution experiment on a tris(oxalate)chromate(III) complex but using modern equipment and much more detailed analysis of the relevant electronic spectroscopic transitions, thereby facilitating application of the most favourable laser wavelengths for irradiating the initially racemic sample to optimize the resulting ee. It is clearly demonstrated that MChPh photochemistry yields higher ees than CPPh on the same system, albeit using a magnetic field of 30T only available in specialized high magnetic field facilities. It is well-written and highly suitable for publication in ACS Central Science.

I have the following minor comments:

1. The caption for Figure 3 repeats that for Figure 2!

2. I wonder if the Nature News and Views article which set the original MChPh report [15] into its historical context should be referenced? (L.D. Barron, Chirality, Magnetism and Light, Nature 2000, 405, 895-896).

3. It might be mentioned (perhaps in the final paragraph) that supernovae generate huge magnetic fields and intense unpolarized light, so could be highly favourable for MChPh enantioselection on organic matter in nearby interstellar dust clouds.

Reviewer: 2

Comments to the Author

This article investigates enantioselective magneto-chiral photochemistry (MChPh) using the tris(oxalato)chromate(III) system. Through magneto-chiral dichroism (MChD) spectroscopy and MChPh experiments, irradiating a racemic solution at 5 °C with 500 mW unpolarized light at 695.5 nm under a 30 T magnetic field for 30 minutes yielded a 0.50% enantiomeric excess (ee). Circularly polarized photochemistry (CPPh) experiments under identical conditions produced lower ee, highlighting MChPh's superior efficiency in enantioselective induction. The study demonstrates a strong correlation between ee and MChD spectral features, with the induced ee increasing linearly with magnetic field strength without signs of saturation. These findings establish a framework for optimizing MChPh through wavelength selection and higher magnetic fields, reinforcing its potential as a competitive mechanism for generating molecular chirality compared to CPPh. Overall, the manuscript is well-written, but the following issues need to be addressed before it can be published in ACS Central Science:

1. The legend for Figure 3 is erroneous and duplicated with that of Figure 2.

2. This manuscript reports a half-life of 35 minutes for the enantiomeric excess (ee) (Figure S8) but does not deeply analyze the competitive relationship between racemization kinetics and the MChPh induction process. Do magnetic fields and light irradiation

simultaneously promote racemization? We suggest supplementing experiments on the dependence of racemization rates on light intensity and magnetic field strength.

3. The light irradiation power is fixed at 500 mW in the study, yet the dependence of ee on power is not explored. We recommend adding power gradient experiments and analyzing the mechanistic correlation between light intensity and ee.

4. This work uses a 0.03 M aqueous solution of potassium tris(oxalato)chromate(III) as the reaction system but does not discuss the influence of solution concentration on the efficiency of magneto-chiral photochemistry (MChPh). Does concentration affect the enantiomeric excess (ee)? Might different concentrations alter racemization rates and thus shift the ee peak value?

5. The Supporting Information should directly provide the molecular structures of (L)- and (D)-tris(oxalato)chromate(III).

6. As mentioned in the manuscript, the MChD signals originate from Boltzmann population differences at low temperatures (MChD C-term) and Zeeman splitting at high temperatures (MChD A-term). These two physical phenomena should be further explained using simpler terms to enhance reader accessibility.

Reviewer: 3

Comments to the Author

In this work, Atzori and coworkers revisit a photoderacemization experiment under magnetic field (up to 30 T), which yields non negligible enantiomeric excess (ee) due to magnetochiral dichroism (MChD). Here this experiment is carried out with a Cr(III) complex offering novel insight into the process. Such studies are relevant as the problem of the emergence of homochirality in the chemistry of life is still an unresolved issue. The work is solid, very well carried out from an experimental point of view and well described. Therefore, I would like to recommend the manuscript for publication in ACS Central Science, pending a few revisions.

1) For their experiments, the authors take mainly advantage of the well-known spin-flip transition of Cr(III), i.e. the 4A2→2E, 2T1 transition (at least in an octahedral-like environment). The authors report that they measure a rather low g_NCD value for this transition. Here, I would like to point out that it may be very difficult to estimate correctly

the Δε_NCD and g_NCD for spin-flip transitions, as the associated ECD and absorption may be heavily overlapped with the higher energy larger bands. Indeed, for suitable compounds, the chiroptical character of spin-flip transitions is better appreciated in emission through CPL. Interestingly a strong chiroptical activity was observed in the very first report of CPL by Emeis and Oosterhof in 1967 with [Cr(en)3]3+, and more recently with more efficient Cr(III) complexes of ddpd and dqp ligands, prepared by the groups of Piguet and Heinze. In fact, the strong g_MChD observed by the authors for this transition would call for a relatively strong m transition moment contributing to the μmm' triple MChD product. The authors may want to provide a little more context to highlight the importance of this transition.

2) The fine structure of the levels involved in the spin-flip transitions may be rather complex in a D2-symmetry environment. Even though a full resolution of all the components may not be feasible, yet some interesting information, perhaps useful for a fuller rationalization of the photoresolution results, may be extracted from MCD and possibly from MChD spectra. For example, a full analysis of magnetooptical data of Cr(III) spin-flip transition was recently reported by Gabbani, Jiménez et al.

3) What is the lowest enantiomeric excess the authors are able to detect in their experimental conditions?

4) "they relative orientation need to fulfil" should read "their relative..."

Author's Response to Peer Review Comments:

Dear Editor,

Detailed point-to-point answers to reviewers and editorial office comments are provided in the attached file.

Yours sincerely,

Matteo Atzori

Associate Editor of ***ACS Central Science***

Please find enclosed the revised version of our manuscript entitled ***"Enantioselective Magneto-Chiral Photochemistry Rediscovered"*** by M. S. Raju *et al.* to be considered for publication in ***ACS Central Science*** as an Article.

First of all, we would like to thank the reviewers for their positive comments and valuable suggestions. In this revised version we have addressed the points raised by the reviewers and we feel that the quality of the manuscript is improved further. Detailed answers to all comments are provided in the following.

***Reviewer 1*: *Publish in ACS Central Science after minor revisions noted.***

***General Comments:*** Magneto-chiral phenomena, which involve a subtle interplay of chirality and magnetism involving unpolarized light propagating collinear with a static magnetic field, are attracting increasing interest both as a new form of chiroptical spectroscopy and for novel technological applications. However, one important aspect, namely magneto-chiral photochemistry for generating an enantiomeric excess, has languished since its first demonstration 25 years ago by one of the authors. Inter alia, this has fundamental relevance for the origin of biomolecular homochirality since it depends on unpolarized light and static magnetic fields which are far more ubiquitous in the cosmos than the circularly polarized light upon which circularly polarized photochemistry (CPPh), currently the most accredited mechanism, depends.

This paper revisits the original MChPh photoresolution experiment on a tris(oxalate)chromate(III) complex but using modern equipment and much more detailed analysis of the relevant electronic spectroscopic transitions, thereby facilitating application of the most favourable laser wavelengths for irradiating the initially racemic sample to optimize the resulting ee. It is clearly demonstrated that MChPh photochemistry yields higher ees than CPPh on the same system, albeit using a magnetic field of 30T only available in specialized high magnetic field facilities. It is well-written and highly suitable for publication in ACS Central Science.

We thank the reviewer for the appreciation of our work.

I have the following minor comments:

**(1)** The caption for Figure 3 repeats that for Figure 2!

We are very sorry with all reviewers for this error. The revised manuscript now contains the correct Figure 3 caption:

"**Figure 3.** Magnetic field (a) and temperature (b) dependence of the D$A_{\mathrm{MChD}}$ signal for (L)-**1** dispersed in a KBr pellet. Insets show the linear dependency of D$A_{\mathrm{MChD}}$ over $\boldsymbol{B}$ and $1/T$ in the investigated ranges (0.0-2.0 T and 4.0-150 K) at l = 696 nm."

**(2)** I wonder if the Nature News and Views article which set the original MChPh report [15] into its historical context should be referenced? (L.D. Barron, Chirality, Magnetism and Light, Nature 2000, 405, 895-896).

DR. MATTEO ATZORI
LABORATOIRE NATIONAL DES CHAMPS MAGNETIQUES INTENSES, CNRS – GRENOBLE
E-mail: matteo.atzori@lncmi.cnrs.fr

According to the reviewer suggestion, the revised manuscript now contains the above-mentioned reference.

**(3)** It might be mentioned (perhaps in the final paragraph) that supernovae generate huge magnetic fields and intense unpolarized light, so could be highly favourable for MChPh enantioselection on organic matter in nearby interstellar dust clouds.

According to the reviewer suggestion, we have now included the following sentence in the Introduction:

"In outer space, huge magnetic fields and intense unpolarized light are generated by supernovae, which provide favourable conditions for enantioselective MChPh on organic matter in nearby interstellar dust clouds."

***Reviewer 2**: Publish in ACS Central Science after minor revisions noted.*

***General Comments:*** This article investigates enantioselective magneto-chiral photochemistry (MChPh) using the tris(oxalato)chromate(III) system. Through magneto-chiral dichroism (MChD) spectroscopy and MChPh experiments, irradiating a racemic solution at 5 °C with 500 mW unpolarized light at 695.5 nm under a 30 T magnetic field for 30 minutes yielded a 0.50% enantiomeric excess (ee). Circularly polarized photochemistry (CPPh) experiments under identical conditions produced lower ee, highlighting MChPh's superior efficiency in enantioselective induction. The study demonstrates a strong correlation between ee and MChD spectral features, with the induced ee increasing linearly with magnetic field strength without signs of saturation. These findings establish a framework for optimizing MChPh through wavelength selection and higher magnetic fields, reinforcing its potential as a competitive mechanism for generating molecular chirality compared to CPPh. Overall, the manuscript is well-written, but the following issues need to be addressed before it can be published in ACS Central Science:

We thank the reviewer for the appreciation of our work.

**(1)** The legend for Figure 3 is erroneous and duplicated with that of Figure 2.

We thank the reviewer for this comment. The revised manuscript now contains the correct Figure 3 caption (see above).

**(2)** This manuscript reports a half-life of 35 minutes for the enantiomeric excess (ee) (Figure S8) but does not deeply analyze the competitive relationship between racemization kinetics and the MChPh induction process. Do magnetic fields and light irradiation simultaneously promote racemization? We suggest supplementing experiments on the dependence of racemization rates on light intensity and magnetic field strength.

The half-life of the ee has been investigated after MChPh experiments without laser irradiation or applied magnetic field. It refers to a thermodynamic, thermally activated, process that bring the unbalanced mixture of enantiomers back to the initial racemic mixture. The effect of only irradiation on racemization is well studied and understood (Stevenson and Verdieck **1968**). Magnetic fields and laser irradiation simultaneously applied promote the generation of the ee through MChPh but do not induce racemization other than that by irradiation alone. The sole presence of a magnetic field, whatever the magnetic field strength, does not produce any ee or affect the racemization. At the same time, the sole unpolarized laser irradiation, whatever the strength, does not produce any ee. Detailed modelling of the process (Raupach *et al. Chem. Phys.* **2000**, *261*, 373-380), taking into account all these aspects, provides a good prediction of the ee, in quantitative agreement with experiments.

**(3)** The light irradiation power is fixed at 500 mW in the study, yet the dependence of ee on power is not explored. We recommend adding power gradient experiments and analyzing the mechanistic correlation between light intensity and ee.

Some laser power dependent experiments were conducted to identify the most effective laser power to be used for the rest of the experiments. In the reported graph is shown the comparison between 30 minutes exposures at $\boldsymbol{B}$ = -15 T and $T$ = 18 ° C with laser powers of 300 and 500 mW at the same irradiation wavelength.

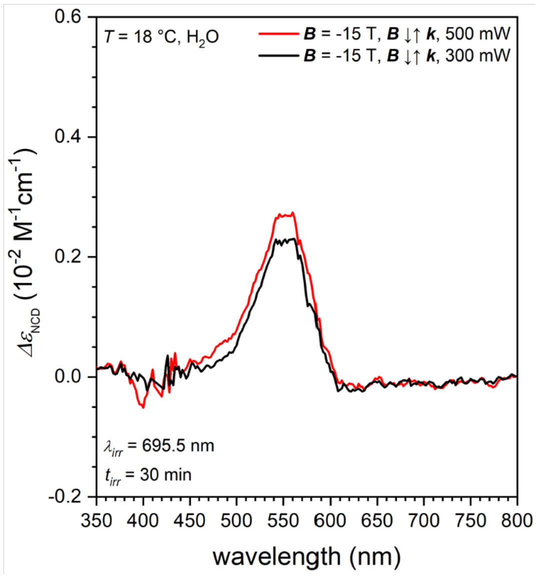


As it can be observed, in the above-mentioned experimental conditions, irradiating with 500 mW provide a slightly higher ee. Higher laser powers were not explored as 500 mW at the end of the irradiating optical fiber was the maximum power that we were able to provide with the available laser source. However, the result shown above shows that the photo-resolution is close to saturation at this power level.

Given the time and budget constraints that are imposed in high magnetic field experiments, we selected 500 mW as laser power for all experiments of the session. This was done to ensure detectable NCD spectra at lower applied magnetic fields and for shorter irradiation times.

**(4)** This work uses a 0.03 M aqueous solution of potassium tris(oxalato)chromate(III) as the reaction system but does not discuss the influence of solution concentration on the efficiency of magneto-chiral photochemistry (MChPh). Does concentration affect the enantiomeric excess (ee)? Might different concentrations alter racemization rates and thus shift the ee peak value?

The reviewer is right; we have not experimentally explored the effect of the concentration on the efficiency of the MChPh. The concentration has been selected previous to high magnetic fields experiments to be the best compromise between the optical absorptions for both spin-allowed and spinforbidden absorption bands of the Cr(III)-based complex. Higher concentrations would result in decreased sensitivity for the NCD measurement on the spin-allowed band whereas lower concentrations would reduce the amount of the absorbed pump light at the spin-forbidden transition. Detailed modelling of the photo-resolution process (Raupach *et al. Chem. Phys.* **2000**, *261*, 373-380) has shown that the relative ee does not depend on the concentration as long as thermal racemization during irradiation is negligible compared to the optically driven process.

**(5)** The Supporting Information should directly provide the molecular structures of (L)- and (D)tris(oxalato)chromate(III).

According to the reviewer request, the molecular structure of the two enantiomers of tris(oxalato)chromate(III) are now reported in the Supplementary Information as new Figure S1.

**(6)** As mentioned in the manuscript, the MChD signals originate from Boltzmann population differences at low temperatures (MChD C-term) and Zeeman splitting at high temperatures (MChD A-term). These two physical phenomena should be further explained using simpler terms to enhance reader accessibility.

According to the reviewer request, we have better detailed the two underlying mechanisms at the origin of the MChD signal at low and high temperature.

The previous text:

"This clearly indicates that at low temperature, the origin of the MChD signals is due to the difference in Boltzmann population of the ground state due to the magnetic field (MChD *C*-term) whereas a *T* = 150 K, the differential absorption arises from the temperature-independent Zeeman splitting of the ground and excited states (MChD *A*-term).[22,23]" Has been modified as follows:

"This clearly indicates that at low temperature, the origin of the MChD signals is due to a difference in
Boltzmann population of the ground state split by the magnetic field (MChD *C*-term).[22,23] The MChD *C*-term, as for the Faraday *C*-term in MCD spectroscopy, is temperature dependent (µ 1/*T*) and has an absorptive lineshape. At *T* = 150 K, the signal has a dispersive lineshape. The dispersive lineshape of the signal at *T* = 150 K is instead indicative that the differential absorption arises from the temperatureindependent Zeeman splitting of the ground and excited states induced by the magnetic field, e.g. a MChD *A*-term, which is the analogous to the Faraday *A*-term in MCD spectroscopy.[25,26]"

***Reviewer 3*: *Publish in ACS Central Science after minor revisions noted.***

***General Comments:*** In this work, Atzori and coworkers revisit a photoderacemization experiment under magnetic field (up to 30 T), which yields non negligible enantiomeric excess (ee) due to magnetochiral dichroism (MChD). Here this experiment is carried out with a Cr(III) complex offering novel insight into the process. Such studies are relevant as the problem of the emergence of homochirality in the chemistry of life is still an unresolved issue. The work is solid, very well carried out from an experimental point of view and well described. Therefore, I would like to recommend the manuscript for publication in ACS Central Science, pending a few revisions.

We thank the reviewer for the appreciation of our work.

**(1)** For their experiments, the authors take mainly advantage of the well-known spin-flip transition of Cr(III), i.e. the $^4A_2 \longrightarrow {}^2E, {}^2T_1$ transition (at least in an octahedral-like environment). The authors report that they measure a rather low $g_{NCD}$ value for this transition. Here, I would like to point out that it may be very difficult to estimate correctly the $\Delta\varepsilon_{NCD}$ and $g_{NCD}$ for spin-flip transitions, as the associated ECD and absorption may be heavily overlapped with the higher energy larger bands. Indeed, for suitable compounds, the chiroptical character of spin-flip transitions is better appreciated in emission through CPL. Interestingly a strong chiroptical activity was observed in the very first report of CPL by Emeis and Oosterhof in 1967 with $[Cr(en)_3]^{3+}$, and more recently with more efficient Cr(III) complexes of ddpd and dqp ligands, prepared by the groups of Piguet and Heinze.

We thank the reviewer for this comment. Indeed, in the submitted manuscript we have highlighted that:

"the $De_{NCD}$ for the $^4A_2$ ® $^2E,^2T_1$ spin-forbidden transition is small (ca. -0.01 $M^{-1}$ $cm^{-1}$) and cannot be easily deconvoluted from the tail of the contribution associated with the $^4A_2$ ® $^4A_1$ transition (Figure 1)." and

"It should be noted that the wavelength dependence of the $g_{NCD}$ shows a minimum at l = 698.5 nm. This is due to a non-negligible absorption coefficient for the $^4A_2$ ® $^2E,^2T_1$ transition and a small NCD response. Therefore, the NCD response of the spin-forbidden band is weak with respect to that of the other transitions."

To take into account the reviewer comment and to better highlight that the determination of a $g_{NCD}$ value for this transition is challenging, the revised version of the manuscript now reads:

"the $De_{NCD}$ for the $^4A_2$ ® $^2E,^2T_1$ spin-forbidden transition is small (ca. -0.01 $M^{-1}$ $cm^{-1}$) and cannot be easily deconvoluted from the tail of the contribution associated with the $^4A_2$ ® $^4A_1$ transition (Figure 1). This introduces an uncertainty in the determination of the $g_{NCD}$ factor (see below). Recent studies have demonstrated that a better estimation of the chiroptical activity of this transition can be obtained by Circularly Polarized Light emission studies.[23]" including the reference: J.-R. Jiménez *et al. Angew. Chem. Int. Ed.* **2021**, *60*, 10095–10102. and

"It should be noted that the wavelength dependence of the $g_{NCD}$ shows a minimum at l = 698.5 nm. This is due to a non-negligible absorption coefficient for the $^4A_2$ ® $^2E,^2T_1$ transition and a small NCD

response difficult to estimate because of the superposition with the tail of the more intense high energy band (see above). However, the NCD response of the spin-forbidden band is weak with respect to that of the other transitions.”

In fact, the strong $g_{MChD}$ observed by the authors for this transition would call for a relatively strong ***m*** transition moment contributing to the ***μmm’*** triple MChD product. The authors may want to provide a little more context to highlight the importance of this transition.

The reviewer is right when he/she highlights the importance of the optical activity of the electronic transition into the MChD intensity. This has been recently experimentally demonstrated for the electronic transitions of lanthanide complexes but not, to the best of our knowledge, for transition metal complexes.

However, it should be noted that the MChD signal observed for the spin-flip transition is indeed stronger than that observed for the spin-allowed transitions but it is not particularly strong compared to other transition metal-based systems previously investigated (see for example *Sci. Adv.* **2021**, *7*, eabg2859). Therefore, the small $g_{NCD}$ observed for this transition is not in disagreement with the observed D$A_{MChD}$ and $g_{MChD}$ values.

To take into consideration the reviewer comment about the features of the investigated transition we have added the following statement in the revised version of the manuscript when describing the nature of the Cr(III) electronic transitions:

The absorption spectrum (350-800 nm) is characterized by three main absorption bands associated with two spin-allowed ($^4A_2$ ® $^4T_1$, $l_{max}$ = 422 nm; $^4A_2$ ® $^4T_2$, $l_{max}$ = 572 nm) and one spin-forbidden ($^4A_2$ ® $^2T_1$,$^2E$, $l_{max}$ 698.5 nm) *d-d* electronic transitions (Figure 1 and S1).[19] These latter transitions, where the excited states differ from the ground state only by the spin multiplicity, are also called spin-flip transitions.[22] and cited the reference: A. Gabbani *et al. Chem. Sci.* **2024**, *15*, 17217-17223.

**(2)** The fine structure of the levels involved in the spin-flip transitions may be rather complex in a D2symmetry environment. Even though a full resolution of all the components may not be feasible, yet some interesting information, perhaps useful for a full rationalization of the photoresolution results, may be extracted from MCD and possibly from MChD spectra. For example, a full analysis of magnetooptical data of Cr(III) spin-flip transition was recently reported by Gabbani, Jiménez et al.

We thank the reviewer for this suggestion. Indeed, the spectral bandwidths of both MCD and MChD spectra does not allow us to analyse the fine structure of the spin-forbidden transitions. We consider that a full analysis of the magnetooptical data as the one reported in the suggested reference is beyond the scope of this article.

**(3)** What is the lowest enantiomeric excess the authors are able to detect in their experimental conditions?

The lowest enantiomeric excess detectable is ca. 0.08 % as can be evidenced by the calibration curve. It is dictated by the detection limits of the commercial NCD spectrophotometer used.

**(4)** “they relative orientation need to fulfil” should read “their relative…” Corrected as suggested.

***Editorial Office Requests/Formatting Changes***

We have also answered to the requests of the Editorial Office as reported below.

**(1).** Please include the email address(es) of the corresponding author(s) on the first page of the manuscript. Done as requested.

**(2).** ACS Central Science requires a brief synopsis. The synopsis should be no more than 200 characters (including spaces) and should reasonably correlate with the Table of Contents (TOC) graphic. The synopsis is intended to explain the importance of the article to a broader readership across the sciences. Please place your synopsis in the manuscript file after the TOC graphic and label as "Synopsis."

The revised version of the manuscript now contains a synopsis which accompanies the TOC graphic.

"Here we report on the Magneto-Chiral Photochemistry of tris(oxalate)chromate(III). Enantiomeric enrichments of 0.50% are obtained irradiating a racemic mixture with a laser beam under magnetic fields."

We hope that the revised version of our manuscript satisfies all requests of the reviewers and editorial office and that it can be now accepted for publication.

Yours sincerely,

**Matteo Atzori**

Matteo Atzori
